# “Precious Time Together Was Taken Away”: Impact of COVID-19 Restrictive Measures on Social Needs and Loneliness from the Perspective of Residents of Nursing Homes, Close Relatives, and Volunteers

**DOI:** 10.3390/ijerph19063468

**Published:** 2022-03-15

**Authors:** Suzie Noten, Annerieke Stoop, Jasper De Witte, Elleke Landeweer, Floor Vinckers, Nina Hovenga, Leonieke C. van Boekel, Katrien G. Luijkx

**Affiliations:** 1 Tranzo, Tilburg School of Social and Behavioral Sciences, Tilburg University, P.O. Box 90153, 5000 LE Tilburg, The Netherlands; s.noten@tilburguniversity.edu (S.N.); k.g.luijkx@tilburguniversity.edu (K.G.L.); 2 HIVA—Research Institute for Work and Society KU Leuven, P.O. Box 5300, 3000 Leuven, Belgium; jasper.dewitte@kuleuven.be; 3 Department of General Practice and Elderly Care Medicine, University of Groningen, University Medical Center Groningen, 9713 GZ Groningen, The Netherlands; e.g.m.landeweer@umcg.nl (E.L.); f.vinckers@umcg.nl (F.V.); n.g.hovenga@umcg.nl (N.H.); 4 Department of Orthopaedic Surgery, FORCE (Foundation for Orthopaedic Research Care Education), Amphia Hospital, 4819 EV Breda, The Netherlands; lvanboekel@amphia.nl

**Keywords:** social contacts, social needs, loneliness, residents, nursing homes, COVID-19 pandemic

## Abstract

During the COVID-19 outbreak in March 2020, restrictive measures (e.g., prohibiting physical visits and group activities) were introduced in nursing homes to protect older residents. Although the importance of social contacts and social activities to fulfill social needs and avoid loneliness is known, these were challenged during the pandemic. This qualitative study specifically focused on how residents, close relatives, and volunteers in nursing homes experienced the restrictive measures in retrospect and gained insights into the impact of the restrictive measures on social needs and loneliness, and the lessons that could be learned. Thirty semi-structured, face-to-face interviews with residents and close relatives, and one online focus group with ten volunteers, were conducted. Recruitment took place at psychogeriatric and somatic units in the Northern, Eastern and Southern regions of the Netherlands and Flanders, Belgium. The interviews and focus group were transcribed verbatim, and an open, inductive approach was used for analysis. Alternative ways of social contact could not fully compensate for physical visits. Generally, participants reported that it was a difficult time, indicated by feelings of loneliness, fear, sadness, and powerlessness. A great diversity in loneliness was reported. The most important reasons for feeling lonely were missing close social contacts and social activities. The diversity in the impact of restrictive measures depended on, e.g., social needs, coping strategies, and character. Restrictive COVID-19 measures in nursing homes resulted in negative emotions and unmet social needs of residents, close relatives, and volunteers. During future outbreaks of the COVID-19 virus or another virus or bacterium, for which restrictive measures may be needed, nursing homes should actively involve residents, close relatives, and volunteers to balance safety, self-determination, and well-being.

## 1. Introduction

At the start of the COVID-19 outbreak, governments worldwide introduced restrictive measures to protect citizens and reduce the transmission of the virus and related deaths. To protect older residents in nursing homes, nursing homes closed their doors to visitors, including close relatives, and most volunteers, also known as the visitors ban. Doors of Dutch nursing homes were closed between 20 March and 24 May 2020 [1], whereas in Flanders doors were closed between 14 March and 17 May 2020 [2]. Mostly, residents were no longer allowed to leave the premises and often they had to stay in their own room. Moreover, group activities were often prohibited, restricting contact with others, such as other residents or volunteers [3].

The restrictive measures implied physical distancing and social isolation, limiting social connection and support. Older people have previously reported the value of social relationships as important requirements for successful aging. They value these relationships, together with engaging in activities as important aspects of good quality of life. When social connections are challenged, as experienced during the COVID-19 pandemic, this may leave social needs unmet and can arouse feelings of loneliness [4,5,6].

Loneliness is often described as a subjective, unpleasant experience because of a perceived discrepancy between the desired amount, frequency, and closeness of social relationships and actual social interactions [7]. Feelings of loneliness are associated with adverse mental and physical health, and increased mortality risk [8,9,10]. Under normal circumstances, loneliness is present in at least one-third of older people, with an even higher proportion among residents of nursing homes [8,11,12,13]. Risk factors that contribute to loneliness among residents of nursing homes are the decline of the social network (e.g., due to loss of partner, health problems, or reduced mobility) [14] and the loss of autonomy and self-determination [15]. Although the importance of social contacts and autonomy of residents is known, the safety and health of residents were prioritized at the expense of social contacts and autonomy of residents.

The restrictive measures contradict the aim of nursing homes to provide person-centered care, where healthcare professionals, in close collaboration with residents and families, customize care according to residents’ abilities, needs, and preferences whenever possible, allowing autonomy and self-identity, and maintaining the independence of residents [16,17]. Close relatives and volunteers can play an integral role in the fulfilment of social needs, as they often are familiar with the wishes and needs of the residents [18,19]. Close relatives can provide emotional and practical support and engage in activities with residents, which may increase well-being and quality of life of residents and close relatives [19]. Volunteers are also essential in nursing homes and may complement and relieve healthcare professionals [20] and provide support in residents’ well-being, e.g., by going for a walk together, organizing other social activities, or lending a listening ear. The visitors ban put the role of close relatives and volunteers under pressure and may have left social needs unmet. Therefore, we aimed to report on the consequences of the restrictive measures from their perspective.

In the past months, many articles have been published on the consequences of the COVID-19 restrictive measures in nursing homes, for example focusing on social contacts [21,22], loneliness, mental health [23], and well-being [24,25]. Data were mainly obtained by quantitative research methods. So far, little attention has been paid to the narratives of those who it concerns the most, namely, residents, and also their close relatives, and volunteers. However, these are important for an in-depth understanding of their experiences, wishes, and needs, and is crucial information to prepare for further outbreaks of COVID-19 or other infectious diseases. Therefore, this qualitative study reported on the impact of restrictive COVID-19 measures from the perspective of residents, close relatives, and volunteers in nursing homes in the Northern, Eastern, and Southern regions of the Netherlands, and Flanders, Belgium. This paper specifically focused on the fulfilment of social needs and related negative emotions, including loneliness.

## 2. Materials and Methods

This qualitative study consisted of semi-structured individual and duo interviews with residents and close relatives, and one focus group with volunteers. This study was reported following the consolidated criteria for reporting qualitative research (COREQ) guidelines [26].

### 2.1. Interviews with Residents and Close Relatives

#### 2.1.1. Participants and Recruitment

Individuals were eligible to participate if they were (1) residents who lived in nursing homes in the Northern, Eastern, and Southern regions of the Netherlands, and Flanders, Belgium at the time of the visitors ban in March 2020, and their close relatives; and (2) capable of verbally communicating in Dutch. Various background variables were considered in the recruitment process to realize a diverse group, e.g., sex, severity and nature of physical and cognitive conditions (i.e., somatic and psychogeriatric units), residents from locations with severe and less severe COVID-19 outbreaks and measures. In this study a nursing home is considered a residential facility that provides 24 h support for people who require assistance with activities of daily living and have identified health and care needs [27].

In the summer of 2020, the project team approached nursing homes to participate in the study. Care professionals of participating organizations identified eligible participants and sent them an information letter explaining the purpose of the study. For residents with dementia, the legal representative, and, when possible, the resident, was informed about the study.

#### 2.1.2. Data Collection

Between November 2020 and January 2021, residents and close relatives were interviewed face-to-face by one of the researchers (E.L., F.V., J.D.W., or S.N.) in nursing homes. One close relative was interviewed online. Researchers wore face masks and respected the social distancing rules and other measures that applied at that time. Residents and close relatives were interviewed in pairs; solo interviews were performed in case one of them preferred so or if commanded by restrictive measures. Residents with dementia were present in a duo interview and answered for themselves where possible; otherwise, close relatives were interviewed as proxies. Interviews were conducted until saturation was reached, aiming for 5–10 interviews per region (Northern and Eastern regions of the Netherlands were combined), for a total of 20–30 interviews.

The interviewers followed a semi-structured interview guide, which was developed in close collaboration with the sounding board group of the project, representing healthcare professionals, policy makers, and implementation and educational specialists. At the start of the interview, demographic information was collected. The interviewer then asked participants an open question to retrospectively reflect on the impact of the restrictive measures from their perspective. Additionally, optional probing questions were asked, related to the following themes: social contacts, fulfilment of social needs, experienced loneliness, positive and other negative consequences of restrictive measures, and resilience. The interview guide is included in Appendix A. The duration of the interviews ranged between 22 and 90 min. Each interview was audio recorded.

#### 2.1.3. Data Analysis

Interviews were transcribed verbatim and pseudonymized. The project team analyzed and coded the transcripts, facilitated by Atlas.ti version 8 software (ATLAS.ti., Berlin, Germany). An open, inductive approach was used for analysis. First, researchers (E.L., F.V., J.D.W., A.S., and S.N.) read and discussed the transcript of the first interview. Afterwards, E.L., F.V., J.D.W., and S.N. performed the coding of the first transcript independently, and results were compared and discussed until consensus was reached on the first set of codes and their description. The same steps were undertaken for the second interview. The code tree was refined accordingly; existing codes were adjusted when needed and newly obtained codes were added when new themes emerged.

The remaining interviews were divided among the five researchers; researchers were responsible for the main coding of the interviews in their own region, and a researcher from a different region performed a check on the coding to increase the inter-researcher reliability. Differences between researchers were discussed during a consensus meeting and researchers agreed on the final coding. All researchers regularly discussed their work during project meetings.

The interviewer sent a member-check to the participants to approve the content and to give them the opportunity to make suggestions for changes or to add any missing information. Most participants agreed with the member-check, and only a few minor details were amended.

### 2.2. Focus Group with Volunteers

#### 2.2.1. Participants and Recruitment

Participating organizations approached volunteers, by means of an information letter explaining the purpose of the study. In the inclusion of volunteers, various background variables were considered, e.g., sex, years of experience, working at somatic or psychogeriatric units, type of work, continuation or cessation of volunteer work during visitors ban. Volunteers were eligible if they had started their volunteer work before the visitors ban in March 2020.

#### 2.2.2. Data Collection

In March 2021, one online focus group with volunteers representing all three regions was conducted via Zoom, a cloud-based videoconferencing platform (https://zoom.us/; accessed on 24 January 2022). The focus group lasted 82 min and was audio and video recorded.

One of the researchers (E.L.) led the focus group, assisted by S.N. and F.V. Topics that were addressed were the experiences of the volunteers and the impact of the restrictive measures on themselves and the residents. Additionally, probing questions were asked related to possibilities of social contact with residents, fulfilment of social needs, experienced loneliness, positive and other negative consequences of restrictive measures, and resilience of volunteers and residents (Appendix B).

#### 2.2.3. Data Analysis

The focus group was transcribed verbatim and pseudonymized. The code tree of the interviews was used as a base for the coding of the focus group (Appendix C). First, the researchers (E.L., F.V., J.D.W., A.S., and S.N.) discussed the transcript. Afterwards, two coders performed the coding independently (A.S., S.N.) and refined the code tree. Results were compared and disagreement was resolved by discussion.

The interviewer sent a member-check to the volunteers, and no corrections were suggested by the volunteers.

### 2.3. Ethics Statement

The Medical Research Ethics Committee Brabant (MREC Brabant) (NW2020-68) and the Ethics Review Board Social and Behavioral Sciences of Tilburg University (ERB) approved this study (RP277). Participants and/or their close relatives received detailed study information and gave written informed consent as needed before participating. Data were safely stored and only the project team had access to the data.

## 3. Results

This section combines the results of interviews and the focus group. Results of all regions were taken together. The presentation of the results is structured as follows: participant characteristics, information on restrictive measures, general description of social contacts, impact of the restrictive measures on social contacts, social needs and negative emotions, reopening of doors, and evaluation of restrictive measures.

### 3.1. Participants

Thirty interviews were conducted: 19 interviews in pairs, and 11 individual interviews, seven with residents and four with close relatives. Ten volunteers participated in the focus group (Table 1).

### 3.2. Restrictive Measures

Restrictive measures slightly differed per nursing home. Overall, participants indicated that, in March 2020, residents were not allowed to leave the premises and, in most nursing homes, they had to stay in their own room. Furthermore, the communal space was closed, and activities, such as playing cards, having coffee or meals with fellow residents, going for a walk with close relatives, or celebrating the holidays together, were often prohibited. All visitors, including close relatives, were prohibited from entering nursing homes. The regulations regarding the admission of volunteers differed. Mostly, volunteers were not allowed, but some of them were allowed to continue their regular work or to support healthcare professionals with non-care-related tasks such as video calling or meal deliveries.

### 3.3. General Description of Social Contacts

The residents participating in this study showed a large diversity in social contacts, both in terms of size and frequency of contacts. Close contacts of the residents generally consisted of close relatives, such as partners and immediate family, fellow residents, healthcare professionals, and volunteers. Before the visitors ban, most residents were visited daily or a few times a week by one or more close contacts. A wider circle of contacts consisted of friends, extended family, and acquaintances; generally, they visited residents occasionally.

### 3.4. Impact of Restrictive Measures on Social Contacts

Residents, close relatives, and volunteers reported that the visitors ban challenged social contacts. In all cases, close social contacts introduced alternative ways to contact residents, such as digital contact (e.g., video calling), window or balcony visits, postcards or packages deliveries, motivational banners outside the nursing home buildings, birthday parties in front of the window, and a resident with dementia received a visit from her dog. According to participants, the duration of the contacts seemed shorter than before. Frequencies of the contacts either increased because close contacts called or visited by the window more often, remained stable, or decreased because of the omission of spontaneous visits or barriers to alternative means of social contact. Contact with the wider circle often diminished or disappeared during the visitors ban. Several residents explained that the prohibition of activities inside and outside the nursing home led to a diminished or complete loss of social contact, and ensured the disappearance of a routine or daily schedule.

Most residents, close relatives, and volunteers appreciated alternative ways of contact. One resident said it was pleasant to see and hear her partner: *“We had been calling on Skype every day […] and I really enjoyed it”*. Some volunteers reported that residents with a limited social network had the opportunity to participate in video calls with them. However, participants experienced several barriers to alternative means of contact, such as being on a higher floor, having no balcony, hearing loss of the resident, and rainy or cold weather, which limited possibilities to keep in contact. A close relative said: *“The nurses took my mother to the balcony, and I was standing downstairs, if I said something, she did not hear me and she did not understand, you couldn’t see them and you couldn’t hear them”*. In addition, they mentioned that they experienced a lack of privacy because of the physical distance: they indicated that an in-depth conversation, which they valued as important, was therefore more difficult. For most residents with dementia, video calling was not an option, because of the loss of speech or because residents did not understand or recognize the person calling, which discomposed them.

### 3.5. Impact of Restrictive Measures on Social Needs and Negative Emotions

Experiences of the impact of the restrictive measures on social needs and negative emotions varied among participants. Overall, residents, close relatives, and volunteers described the visitors ban as a difficult time, indicated by feelings of loneliness, fear, sadness, and powerlessness. However, a great diversity in loneliness was found. Several residents and partners indicated that they had been lonely: “*It was a very difficult time. I felt even more alone, that’s for sure”* (Resident). Others reported no feelings of loneliness: *“I did not experience loneliness”* (Resident). Loneliness was reported to a lesser extent among other close relatives and volunteers. For some residents having more advanced dementia, the residents themselves or their caregivers found it difficult to say whether they have been lonely.

Residents who experienced loneliness reported several reasons for feeling alone. All stated that they primarily missed close social contacts and being with their relatives or others. They explained that their relatives were the most important people they live for, and, for example, they missed a hug from their children and/or grandchildren. Another reason residents mentioned for feeling alone was they had been alone in their room for a large part of the day and there was not much to look forward to, i.e., no visits and no activities with others. Due to the strict measures, some residents compared the circumstances with being in prison and some referred to World War II. Among the close relatives, we noticed that especially the partners of residents with a smaller social network experienced loneliness, which was enhanced because they could not visit their loved ones living in the nursing home. In contrast, some residents did not feel the need for social contact or activities or were satisfied with the alternative ways of contact and did not feel lonely during the visitors ban.

In addition to loneliness, fear was reported as a negative emotion. Some residents, close relatives, and volunteers experienced fear of becoming infected or infecting others with the COVID-19 virus, especially at the beginning when much was unknown about the virus. Others did not experience any fear or did not think about it; a resident said: *“I was not afraid of the virus, but my children were”*. The impact on residents was large when close neighbors or friends from the nursing home passed away because of a COVID-19 infection.

Close relatives and volunteers stated that the restrictive measures had an emotional impact on them. A partner said: *“It was tough, because I used to visit my wife almost every day”*. Another partner reported that he has been married for 57 years and he had to meet his wife behind the fence, which made him feel very emotional. Close relatives reported sadness and concerns as they were deeply touched by the situation of the residents. They could only observe the situation from a distance and felt like they could not support the residents properly, which made them feel powerless. For volunteers, it was difficult that they could not organize any activities and could not be meaningful for the residents. Volunteers noticed that they felt the need to stay in touch with residents, especially volunteers working one-on-one, but this was not always possible to achieve:* “I was not allowed to enter as a volunteer and I had no contact with the residents at all, that made me very anxious”*. Close relatives of residents with dementia said it was difficult that they were not allowed to visit for such a long time, and that residents sometimes deteriorated in mental functioning and had difficulties recognizing loved ones.

The impact of the restrictive measures varied among residents, close relatives, and volunteers, depending on several factors, such as character (e.g., being a social person, ability to accept the situation), social needs and environmental factors, such as in the case of health professionals. Some residents described themselves as social persons, who like being around other people; they reported being more affected by the restrictive measures than others. Residents that could easily accept the situation were less affected than others. A large social network was described as helpful but could not always prevent loneliness in residents. Residents noticed that the quality of social contacts was more valuable than the quantity; for example, one of the close relatives said: *“I am convinced that family and friends are very important, but when it comes down to it, at times when you [the resident] were having a really hard time, then your closest ones were the most important. So, me and the kids, that’s what you needed the most”*. Overall, residents and close relatives reported that healthcare professionals tried supporting them as much as they could. Although their time was limited, residents appreciated the support with alternative ways of contact, healthcare professionals shortly opened the windows or brought residents outside for a talk with their partner.

### 3.6. Reopening of Doors

After doors reopened in May 2020, one or more visitors were allowed per day (often within a specific timeslot), activities in the nursing homes started again, and meals were served in the communal space, although the 1.5-m distance rule continued to apply. Residents and close relatives reported the moment they saw each other for the first time after the visitors ban as being emotionally laden. They reported that they realized how much they missed each other’s physical presence. For some it was hard to comply with the 1.5-m distance rule and they were not able to resist giving each other a hug or kiss. A close relative said: *“...Then we were allowed to be together again, but we were not allowed to touch each other. It was such an emotional moment, we just had to give each other a hug”*. Gradually, residents and their close relatives were allowed to do activities together, such as going for a walk or taking the resident home, which they both enjoyed immensely. Residents indicated that they immediately felt less alone because they could see their close relatives in person. Moreover, in some cases, digital contact continued in addition to physical visits, as it was evaluated as a positive way to keep in touch, especially for those relatives that lived far away.

All participants experienced the easing of restrictive measures as positive, but some disadvantages of remaining restrictions were also experienced. Social contacts were still challenged because the number of visitors was limited, so, for example, grandchildren could not visit in the first period after the visitors ban. Moreover, limited visiting hours restricted spontaneous visits from those who normally stop by for a cup of coffee and a chat. Visitors and volunteers were still concerned about safety and were aware of the risk of bringing the virus into the nursing home. Some partners and volunteers felt vulnerable because of their age, due to which they were sometimes hesitant to re-enter the nursing home.

### 3.7. Evaluation

Especially at the beginning of the pandemic, residents, close relatives, and volunteers understood the strict measures. The situation sometimes strengthened the relationship between residents and close relatives: a resident put forward the recognition and appreciation of social contact as a positive side of the measures: *“People appreciate more what they have”*. On the other hand, it had been a difficult time. A resident said:* “Life can be over any day. This pandemic is taking away precious time that we could have enjoyed”*. Residents are in their last phase of their lives, and time is precious when living in a nursing home, time she would have wanted to spent with close relatives or fellow residents. An important piece of advice from residents, close relatives, and volunteers was to pay more attention to the social aspect to limit negative consequences of restrictive measures (e.g., in terms of loneliness), and consider their wishes and needs.

## 4. Discussion

Restrictive COVID-19 measures limited social contacts of residents, close relatives, and volunteers in nursing homes in the Netherlands and Flanders. Alternative ways of contact were helpful but could not fully compensate for physical visits. It was a difficult time for residents, close relatives, and volunteers, as indicated by feelings of loneliness, fear, sadness, and powerlessness. A great diversity in loneliness was reported, with missing close social contact and being together with relatives as the most important reasons for feeling lonely. The diversity in the impact of restrictive measures, depended on, e.g., social needs, coping strategy, and character.

To respond to the COVID-19 outbreak, physical distancing and social isolation measures were used in nursing homes to protect residents and reduce the transmission of the virus. Social contacts were challenged because of this; for example, alternative ways of contact were introduced, the duration of contact seemed shorter, and frequency changed. Residents mentioned that the quality of social contacts was more valuable than the quantity, which is in line with previous research [15]. The greatest impact was on residents who are usually socially active, while some residents did not feel the need to socialize and preferred being alone, which is consistent with findings in the general population [28].

Alternative ways of social contact were introduced in all residents and were generally experienced as positive. Especially for close relatives who live further away, digital contact was experienced as a good alternative to contact the resident. The COVID-19 pandemic has recognized the value of technology for communication, which can mitigate the negative effects of social distancing [29]. In other studies, familiar methods of communication, such as phone calls, emails, or written letters, were also reported as beneficial ways of communication that positively influenced the emotional well-being of residents during the restrictive measures regarding in-person visits [30]. Despite the positive experiences with technology, we noticed in our study that it could not fully compensate for physical visits, which is consistent with observations of healthcare professionals [31]. Moreover, digital contact has often not continued after the reopening of nursing homes, because of preferences of physical attendance, barriers to alternative means of contact, and limited time of healthcare professionals to assist residents in using digital contact. A recent study in France reported that residents of nursing homes were able to complete telephone calls more independently than video calls and therefore tended to use the phone more often than video calls. Interestingly, when residents received assistance to establish video calls, they were more satisfied with the use of video calls to communicate with relatives than to use telephone calls [32]. Given this, we recommend healthcare professionals, volunteers, and close relatives adopt user-friendly digital technology if possible. Digital contact cannot replace face-to-face contact [33], but it can complement physical visits, as it increases frequency of contact, facilitates social connections, and may help to alleviate loneliness among residents of nursing homes [34,35]. Based on our results, which show that digital contact is not a good fit for all residents, we also recommend thinking about familiar communication methods for others. In accordance, previous research reported that contact with healthcare professionals can have a great impact on reducing loneliness, as short conversations appeared to be a powerful tool, showing dignity and respect for the residents [36]. In a future situation where doors need to close, several communication methods could be deployed to improve social connectedness between residents and others inside and outside the nursing homes.

In most nursing homes, the communal space was closed, and social gatherings and group activities were often prohibited, restricting contact with other residents and increasing isolation. Other studies have also reported the relevance of activities, which are usually important aspects of residents’ daily routine and are considered important highlights [37]. Participating in organized activities gives residents in nursing homes the feeling of belonging to a group, creating stability and a meaningful environment, and seemed to reduce loneliness [15,36]. The loss of activities and social contact were reported as the most important reasons for sadness and loneliness during the visitors ban in nursing homes [31], as these result in a lack of daily structure and meaning in life. Therefore, it is recommended to try to continue to organize social activities for residents in nursing homes, whenever possible, in a safe manner, e.g., by assigning residents to bubbles for activities while keeping a safe distance.

Unfulfilled social needs for meaningful relationships have resulted in negative emotions and feelings among residents, close relatives, and volunteers. Our results match those observed in earlier studies, where high levels of sadness, anxiety, and loneliness were found among residents of nursing homes in the Netherlands [25,31] and Belgium [37,38] during the period of COVID-19. Previous research also underscored difficulties close relatives experienced coping with anxiety regarding safety of residents [39]. Our results on loneliness broadly support quantitative data obtained in Dutch nursing homes, which reported loneliness among the majority (77%) of residents during the first lockdown in May 2020 [25]. A longitudinal study among Dutch older adults reported an increase in loneliness during the pandemic (May 2020) compared to 7 months earlier [23]. In accordance with our results, older adults reported missing close connectedness and having people around them. In our study, loneliness of residents was reduced the moment physical visits were allowed, which indicates the importance of being together. Interestingly, another follow-up study in the United States reported a slight increase in loneliness in older adults after the initiation of social distancing measures, which levelled off thereafter. The authors suggest this could be a result of the absence of social connections due to the measures, which was resolved over time as participants perceived more social and emotional support in other ways than in person [40]. In accordance with our study results, this showed the importance of social connections to decrease loneliness.

In this study, important information was provided on the impact of the restrictive measures from the perspective of residents with dementia. For those with more advanced dementia, information was limited because they had difficulties expressing themselves. During the interviews, close relatives could support those residents in recalling memories, but they found it difficult to say how residents experienced the restrictive measures, as they could only observe from a distance. Because alternative ways of contact are often not well understood by residents with dementia, healthcare professionals should identify possibilities and adhere to preferences of those residents and close relatives to stay in touch with each other, which was underlined in previous research [39].

Three Dutch studies that included the perspective of healthcare professionals reported that residents without or with mild cognitive impairments were more affected (e.g., high levels of loneliness, depression, and behavioral problems) by the restrictive measures than those with more advanced dementia [25,35,39]. Research has also shown some positive consequences. For residents with dementia, a decrease in agitation, aggression, and wandering was found [31,35]. The decrease in challenging behavior could be a result of a reduction in overstimulation. The lives of residents were more quiet because visitors were not going in and out all the time, but it is important to offer sufficient stimuli to limit apathy.

The restrictive measures contradict the aim of nursing homes to provide person-centered care. The individual differences in the impact of the restrictive measures on residents confirmed the importance of person-centered care. In person-centered care, healthcare professionals, together with residents and close relatives, try to adhere to the needs and preferences of residents whenever possible [16,17], allowing autonomy and self-identity, and maintaining the independence of residents [41].

During the visitors ban, healthcare professionals did not always have the time, and close relatives and volunteers could not provide emotional and practical support. The wishes and needs of residents and close relatives were not included in the policy making and little attention was paid to their autonomy [42]. In a recent study, healthcare professionals reported that the highest value should be placed on the autonomy of older people to achieve successful person-centered care that respects their values [43]. The loss of autonomy and self-determination was also mentioned as contributing to loneliness [15]. From the perspective of healthcare professionals, this COVID-19 period has confirmed the added value of close relatives and volunteers in nursing home care [31], who contribute to maintaining the quality of life of residents [19,20], specifically, because they are familiar with the personality, wishes, and needs of the residents.

Research on the reopening of doors has shown positive effects on residents’ well-being and quality of life [24,44,45], showing the importance of allowing at least one visitor at all times. To limit risks of allowing visitors during an outbreak, nursing homes should prepare for a tailored approach that fits residents, close relatives, volunteers, and health professionals, including testing and tracing, special COVID-19 units for quarantine, and personal protection equipment. Although regulations have changed in the meantime, the organizational workload has endured, and dilemmas have remained.

### 4.1. Strengths and Limitations

The current study had several strengths, including the qualitative study design with interviews and a focus group, and the inclusion of residents, close relatives, and volunteers in different regions with different measures and severities of COVID-19 infections. There are also a few limitations to note. First, this study aimed to perform interviews in pairs, including residents and their close relatives; as a result, residents without close social contacts were not included, which might have resulted in an underestimation of reported loneliness in our study. Since many residents never receive visitors (one in eight nursing home residents in The Netherlands [46]), further research on loneliness should specifically aim to include those. Second, it is important to bear in mind that the interviews and focus group took place at least six months to a year after the complete visitors ban, which might have affected the results. Although we believe that most residents could recall the feelings regarding the visitors ban because some restrictive measures were still present at the time of the interviews and the focus group, the passage of time and changes in regulations in nursing homes and in society, and the social perspective, could have faded away or influenced their memories.

### 4.2. Lessons Learned

Protecting older people in nursing homes from the COVID-19 virus was important, but considering the negative impact of restrictive measures, it is recommended to find a balance between safety, self-determination, and well-being, following the person-centered care approach. Nursing homes should effectively respond to infectious disease outbreaks using infection control practices, while collaborating with residents and close relatives to meet the social needs and limit loneliness, which should be encouraged by the government. As the importance of social contact and activities for residents of nursing homes is recognized, the commitment of volunteers and close relatives should be considered. Based on the diversity of experiences found in this study, we recommend nursing homes to adhere to person-centered care and individual decision making as much as possible, while weighing risks associated with each decision. Lessons learned from this study have immediate relevance and can be used to plan and prepare for further outbreaks of COVID-19 or other infectious diseases.

## 5. Conclusions

Restrictive COVID-19 measures in nursing homes resulted in negative emotions and unmet social needs of residents, close relatives, and volunteers. During future outbreaks of the COVID-19 virus or another virus or bacterium, for which restrictive measures may be needed, nursing homes should actively involve residents, close relatives, and volunteers to balance safety, self-determination, and well-being.

## Figures and Tables

**Table 1:** Participants’ characteristics.

Residents	
Age range	57–101 years
Sex	
Female	23
Male	7
Unit	
Somatic	12
Psychogeriatric	5
Mixed	13
COVID-19 infection	11
**Close relatives**	
Daughter	12
Partners	7
Son	2
Daughter-in-law	1
Brother	1
**Volunteers**	
Age range	59–76 years
Sex	
Female	8
Male	2
Years of volunteer work in nursing home	2–14 years
Volunteer work during visitors ban	
Yes	3
No	7

## Data Availability

Data is contained in the Appendix C.

## Appendix A

**Table A1:** Semi-structured interview guide for interviews with residents and close relatives.

Topics	Probing Questions
General experiences of visitors ban	How did you experience the visitors ban? How did the visitors ban affect you? How did you deal with the visitors ban? What was helpful for you?
Daily activities	Could you tell me how you usually spend your days? How has this changed during the visitors ban? How did that make you feel? How did you deal with this? What was helpful for you?
Social contacts and social needs	Could you tell me about your social contacts? What do you think about your social contacts? What were your social contacts like during the visitors ban? How did that make you feel? What was helpful for you?
Loneliness	Do you have a close or intimate relationship with someone? Do you have as many social contacts as you would want to have? To what extent can you still do what is important to you? Have you felt lonely or sad during the visitors ban? At the moment, do you still experience these emotions?
Other (positive) consequences of restrictive measures and relaxation	Are there other things that you have experienced or that you think are important to mention about the visitors ban? What were the positive aspects of not being allowed to receive visitors? What do you think of the other measures that you and others had to deal with? Are there any aspects that you find positive? Are there any changes that you would like to keep, for yourself or others? Could you mention anything that you would have done differently or things that should change?

## Appendix B

**Table A2:** Semi-structured interview guide for focus group with volunteers.

Topics	Probing Questions
Changes in volunteer work during visitors ban	Were there any changes for you as a volunteer? Could you continue your work as a volunteer? In case volunteers could not continue their volunteer work: Were you able to keep in touch with residents?
General experiences of visitors ban	How did you experience the visitors ban? How did the visitors ban affect you?
Loneliness	Have you felt lonely or sad during the visitors ban? How did you deal with this? What was helpful for you? Have you experienced loneliness or sadness among residents? How did you deal with this?
Evaluation of visitors ban	Do you feel the visitors ban was justified? Could you mention anything that you would have done differently or things that should change? Do you have any advice or lessons learned for the future?

## Appendix C

**Table A3:** Code tree of interviews and focus group.

Main Themes	Subthemes
Background information	Unit size
	Unit type
	Employment close relative
	Employment volunteer
	Duration volunteer work
	COVID infection resident
	COVID infection close relative
	COVID infections nursing home
	COVID cohort section
	Duration stay in nursing home
	Health resident
	Age resident
	Age close relative
	Age volunteer
	Relationship close relative and volunteer
	Personality resident
	Personality close relative
Social contacts	Marital status
	Frequency contact
	Size network
	Negative appreciation contact
	Positive appreciation contact
	Reciprocity
	Loss of contact resident with close relative
	Loss of contact resident with fellow residents
	Loss of contact resident with volunteer
Activities resident	Activities in nursing home
	Computer use
	Closing communal space
	Reading
	Meals
	Listening to music
	Stay in their own room
	Other activities
	Watching television
	Walking
	Cancellation of activities
	Ability to keep yourself busy
Activities close relative	Closing communal space
	Other activities
	Loss of daily activities
	Doing laundry and groceries for resident
	Cancellation of activities with resident
	Cancellation of activities
Activities volunteer	Tasks volunteer work
	Loss of tasks volunteer work
	Returning tasks volunteer work after lockdown
	Continuing volunteer work during lockdown
	Frequency volunteer work
Actions resident	Acceptance of situation
	Balcony or window visit
	Video calling
	Sending letters
	Reading
	Other ways of contact
	Other actions resident
	Other hobbies
	Partners support each other
	Religion
	Support of fellow residents
	Calling
	Watching television
	Walking
	To isolate oneself from others on difficult moments (e.g., staying in bed)
Actions close relative	Acceptance of situation
	Organising activities for resident
	Balcony or window visit
	Video calling
	Sending letters
	Mobilizing social contacts
	Reading
	Encourage resident
	Other ways of contact
	Other actions close relative
	Other hobbies
	Sending packages
	Partners support each other
	Religion
	Spreading out contacts
	Calling
	Watching television
	Walking
Actions volunteer	Outdoor visitation (e.g., in shipping containers)
	Video calling
	Calling
	Sending letters
	Sending packages
Policy nursing home	Actions staff
	Policy nursing home
	Communication
	Considering wishes and needs of residents, close relatives and volunteers
	Aftercare
	Understaffing
	Limited time staff
	Volunteers
	Appreciation staff
Barriers	Regulations visitors
	Uncertainties about the virus
	Being on a higher floor
	Other barriers
	Deaths
	Hearing loss
	Change of structure of department
	Loss of practical support by close relative
	Loss of supervision by close relative
Negative emotions resident	Fear
	Loneliness
	Skin hunger
	Decreased vitality
	Difficult time
	Powerlessness
Negative emotions close relative	Fear infection or death resident
	Loneliness
	Skin hunger
	Difficult time
	Powerlessness
Negative emotions volunteer	Fear infection or death resident
	Fear infection volunteer
	Loneliness
	Skin hunger
	Difficult time
	Powerlessness
Positive emotions resident	Positive emotions
Positive emotions close relative	Positive emotions
Positive emotions volunteer	Positive emotions
Dementia	Deterioration of functioning
	Not noticing restrictive measures
Reopening of doors	Reopening of doors
Evaluation of measures	Advices
	Understanding of measures
	One visitor is important
	Missing perspective
	No recognition elderly
	Positive and negative consequences visitors ban
	Involvement of volunteers versus close relatives
	Vaccinations: hope
	Norms
	Values
Other	Impact restrictive measures for couples
